# *Origanum majorana* Essential Oil Inhalation during Neurofeedback Training Reduces Saliva Myeloperoxidase Activity at Session-1 in Bruxistic Patients

**DOI:** 10.3390/jcm8020158

**Published:** 2019-01-31

**Authors:** José Joaquín Merino, José María Parmigiani-Izquierdo, María Elvira López-Oliva, María Eugenia Cabaña-Muñoz

**Affiliations:** 1 CIROM Center, Centro de Implantología and Rehabilitación Oral Multidisciplinaria, 30001 Murcía, Spain; jmparmi@clinicacirom.com (J.M.P.-I.); mecjj@clinicacirom.com (M.E.C.-M.); 2 Sección Departamental de Fisiología, Facultad de Farmacia, Universidad Complutense de Madrid (UCM), 28040 Madrid, Spain; elopez@farm.ucm.es

**Keywords:** stress and brain plasticity, brain stimulation, neuromodulation, bruxistic patients and aromatherapy, Origanum majorana essential oil, nasal filters (activated carbon), myeloperoxidase, neuroscience, Neurofeedback, brain research

## Abstract

**Introduction:** Bruxism affects teeth and provokes sleep alterations. We evaluated whether saliva Myeloperoxidase (MPO) activity could be reduced after 21 neurofeedback training (NO) sessions in *Origanum majorana* (*AE*) bruxistic-treated patients (*n* = 12 patients, 120 saliva samples). The term divergence evaluates cerebral activity, which was compared between bruxistic patients with/without this essential oil exposure during 21 NO training sessions (*n* = 12, *n* = 120 saliva samples). The nasal filter used allow us to vehiculizate this *Origanum majorana* essential oil in patients. MPO activity was measured in six training NO sessions (Session S1, 6, 12, 18, 21). We included a total of 104 patients and 500 saliva samples. **Results:** there was a fast reduction in MPO activity after NO session-1 in bruxistic patients; divergence (an index of NO training brain efficacy) is the difference in cerebral activity found between pre-training and post-training levels. Thus, Divergence can fluctuate during NO training before reaching a final calm state after many sessions (21). Repeated NO training lead to habituation in so far as cerebral activity as well as MPO activity after 21 training sessions. *Origanum majorana* essential oil modulates cerebral activity at certain training sessions in bruxistic patients. Stress levels were reduced on the perceived stress scores (PSS: Cohen Scale) after 21 NO sessions than for those bruxistic without NO training sessions. **Conclusions:** MPO activity could predict stress level in bruxistic patients and repeated NO reduced their stress level; *Origanum majorana* essential oil enhanced these anxiolytic effects.

## 1. Introduction

Recently, Lobbezzo et al., 2018 defined bruxism as follows: “Bruxism is a repetite jaw-muscle activity characterized by clenching or griding of the teeth and/or by bracing grinding or thrusting of the mandibule” [1]. Bruxim has two distinct circadian manifestations because it can occur during sleep (sleep bruxism, SB) or during wake fullness (awake bruxism) [1]. Bruxism is mainly regulated centrally, not peripherally [2]. Bruxism is affected by stress or other anxious conditions, which increase the frequency of episodes [3]. Bruxism has been associated with several factors, including daytime stress, biopsychosocial anxiety, and obstructive sleep apnoea [4,5,6,7,8,9,10,11,12]. SB decreases with age, without any gender difference. The prevalence of regularly reported SB was established at 8.6% on a total of 6357 patients in Canada [13]. It has also been hypothesized that bruxism is part of a sleep arousal response and is modulated by various neurotransmitters in the central nervous system. Similarly, disturbances in the central dopaminergic system have been linked to bruxism [5]. Several therapeutic treatments have been proposed, especially strategies involving drugs that regulate dopamine metabolism or benzodiazepines treatment [14,15,16,17]. The World Health Organization (WHO) recommends the use of phytoteraphy [18,19,20,21,22]. However, it is necessary to investigate new biomarkers that could predict stress behaviour in these bruxistic patients as myeloperoxidase activity (MPO).

MPO is a glycosylated heme enzyme, which is isolated from polymorphonuclear neutrophils and macrophages. MPO activity contributes to the propagation of oxidative stress in acute inflammatory conditions [23,24]. Saliva MPO activity can predict the severity of periodontal disease since is considered a gingivitis marker in patients [25].

The aromatherapy field emerges as a safe and economic alternative to prevent emotional alterations in patients [26]. *Origanum majorana* L. (*O. majorana*) belongs to the Lamiaceae family, and contains several terpenoids, that are isolated from aerial parts of the Origanum plant and exhibit antimicrobial, antiviral, antioxidant properties without toxic effects [27,28]. The antioxidant potential properties of *Origanum dictamus* essential oil is associated to the constituent carvacrol (52%) or gamma-terpinene compounts (8.4%) [29,30]. The *Origanum majorana* essential oil used here contains terpenoid oils with antioxidant properties (alpha-terpinene 14.10%, gamma terpinene 14.1%, Cis Tuyhanol 15.2) as well as minoritary constituents (<0.01%). In folk medicine, *Origanum majorana* is used for cramps, depression, migraine, nervous headaches [27] since emotional responses can be modulated by different odors.

Electroencephalography (EEG) records neuron electrical activity in the form of brain waves [31]. The EEG can be differentiated into delta wave (0.5~3 Hz), theta wave (4~7 Hz), alpha wave (8~12 Hz), SMR (sensorimotor rhythm) wave: 13~15 Hz), low beta wave (16~20 Hz), high beta wave (21~40 Hz) depending on each frequency. Global neurofeedback technology (also termed NeurOptimal, NO) is a new version of neurofeedback that measure the total cerebral activity in patients; NO is a non-invasive self-training technology that improves brain functioning [11] as a consequence of overtraining by regulating waves in the prefrontal cortex or cingulate cortex (cerebral activity) of patients [21,31,32]. After neurofeedback training, patients change their brain wave activity in real time during each NO training session. During a NO session participants are listening a classic piece of music (the same for all of them) given the beneficial effect of music for patients [33,34,35]. NO training uses “music interruptions” to identify changes in brain activity. Local and inhibitory/excitatory interactions shape neuronal representations of sensory, motor, cognitive variables, and produce local changes on electroencephalographic (EEG) gamma frequency (30–80 Hz) oscillation [33,34,35]. Alpha and beta waves can detect different emotional responses in patients. A beta wave represents a fast wave and is associated with high arousal, concentration and focused attention after Neurofeedback training in participants [35,36]. However, NO training indicates the global waves of cerebral activities during each session. Lineal neurofeedback training leads to beneficial effects on working memory, attention, and cognitive processes. Up until now, all neurofeedback-based research on emotion has been performed on healthy participants and patients with brain disabilities and central nervous system pathologies [36,37,38,39,40,41].

### Aim

- We evaluate whether NO training in 21 consecutive training sessions could decrease cerebral activity (divergence) in bruxistic patients with high “intrinsic stress” and whether smelling *Origanum majorana* essential oil during NO training affect results.
- We evaluated whether *Origanum majorana* essential oil (1% impregnated into nasal filter) could regulate their saliva MPO activity during 21 NO training sessions by measuring every six the NO sessions, including the first and last of 21 sessions (MPO: S1, 6, 12, 18, 21) in bruxistic patients exposed to this fragrance in 21 NO sessions and those NO trained bruxistic patients not exposed to this essential oil.
- We also evaluated whether *Origanum majorana* essential oil inhalation during 21 NO training sessions could reduce stress symptoms in bruxistic patients by decreasing their PSS (stress perceived scale) scores as compared to unexposed bruxistic patients to this fragance during 21 NO sessions.

## 2. Materials and Methods

### 2.1. Colorimetric Assay for Saliva Mieloperoxidase (MPO) Activity

Saliva samples were collected at the morning (09.00–11.00 am) before/after NO training in patients that visit a dental clinic (CIROM); they do not have brain pathology or behavioural problems. The food intake was not allowed for the subjects at least 90 min before collecting saliva samples for MPO activity determination. Saliva MPO activity were evaluated by a colorimetric assay using DAB as substrate [42]. 3,3′-Diaminobenzidine tetrahydrochloride (DAB) and hydrogen peroxide (H_2_O_2_) were purchased from Sigma-Aldrich (Spain). All chemicals used here were analytical grade. Saliva samples (100 μL) were pipetted into 1 mL of the 0.5 mM DAB solution (0.9 g DAB in 50 mL of 0.1 M potassium dihydrogenphosphate pH 4.5. Fifty μL of 6 mM H_2_O_2_ were added to initiate the reaction. After incubation at room temperature for 20 min, 20 μL of 0.1 mM sodium azide has been added to stop the reaction. Absorbance was measured at 465 nm (Thermo Scientific, Madrid, Spain).

### 2.2. Origanum Majorana Essential Oil

Patients smelled 1% *Origamum majorana* essential oil (PRANAROM^®^, Barcelona, Spain), which was impregnated into nasal filters (activated carbon, Inspira-Health^®^, Barcelona) by diluting with neutral essential oil. This neutral oil, OF16850, PRANAROM^®^) was used as control-placebo in patients. Participants smelled 1% *O. majorana* essential oil impregnated into nasal filter during all NO training sessions. The major volatile constituents of *Origanum majorana* essential oil detected by chromatography are tuyhanol and terpenes oils in these percentage: (alpha-terpinene 14.10%, gamma terpinene 14.1%, terpinolene 3.17%, Trans-Thuyanol 3.44%, terpinnene 4-oil 23.6%, alpha-Terpineol 3.1%, Cis Tuyhanol 15.27%, alpha Therpineil 3.1%, Sabinene 8.27% and other minoritaries in a proportion less than 0.01%). 

### 2.3. Sample Analysis

All procedures, including the writing informed consent forms were conducted following the ethical standards of the Helsinki Declaration of 1975 (revised in 2000). This study has been approved by the Centro de Implantologia and Rehabilitación Oral Multidisciplinaria (CIROM) research commitee (#2015-02, Murcia). All subjects were properly instructed and signed the appropriate informed consent form. All efforts were made to protect patient privacy and anonymity. The CIROM has been approved and certified by AENOR Certification and Normalization Spanish Agency (Spain; CIROM CERTIFICATE for dentist services; Directives CD-2014-001 number; ER-0569/2014, UNE-EN ISO 9001: 2008 and UNE 179001-2001, Spain, Europe). All patients have been selected according to these inclusion criteria. They are living in Murcia (Spain, Europe). They are 45 years old (average) and their sociocultural status are medium/high; bruxistic participants with “intrinsic stress” as well as a good general healthy state were selected in the present study; they visit a dental clinic (CIROM) for routine evaluation. The Depressive Stress, Anxiety Scale-42 as well as a bruxism test identified bruxistic patients with “high inherent stress” and controls (without stress) according their reached scores in the Stress item (DASS-42). These bruxistic participants filled up the Perceived Stress Scale (PSS) before/after 21 NO training sessions (8 a.m. to 14 p.m.). All patients were naive to NO technology and they never participated in neurofeedback/NeurOptimal studies before. NO sessions were conducted by an experienced trainer (MEC) and participants were trained during 21 sessions (2 sessions/week). The female percentage is 55%.

#### 2.3.1. Study Groups: Design

The total number of enrolled participants is 104 and MPO activity was evaluated in 500 saliva samples by enzymatic assay (see materials and methods). The experimental design includes: (a) trained bruxistic patients (with high “intrinsic stress”) during 21 Neurofeedback sessions (NO) who smell *Origanum majorana* essential oil in each training session (*n* = 12; 120 saliva samples); (b) bruxistic participants (with higher “intrinsic stress”) were trained during 21 NO sessions without smelling the fragance (*n* = 12, 120 samples). We also included (c) bruxistic participants (with high “intrinsic stress”) who were not trained in NO (*n* = 30, *n* = 120 saliva samples) as well as (d) 5 trained controls that were non bruxistic participants (without stress) who received NO training during 21 sessions (*n* = 5, 50 saliva samples). Finally, (e) 30 untrained NO controls (without stress) were also included here (*n* = 30, 60 saliva samples). All saliva samples were collected between 8–11 a.m. 

We also included control subjects that smelled *Origanum majorana* essential oil. They underwent 12 NO sessions exposed to *O. majorana* essential oil (*n* = 5, 15 saliva samples). The control placebo group (without stress) smelled a neutral oil during 12 NO sessions. They underwent 12 NO sessions exposed to *O. majorana* (*n* = 5, 15 saliva samples, see Table 1 and Table 2).

**Figure 1:**
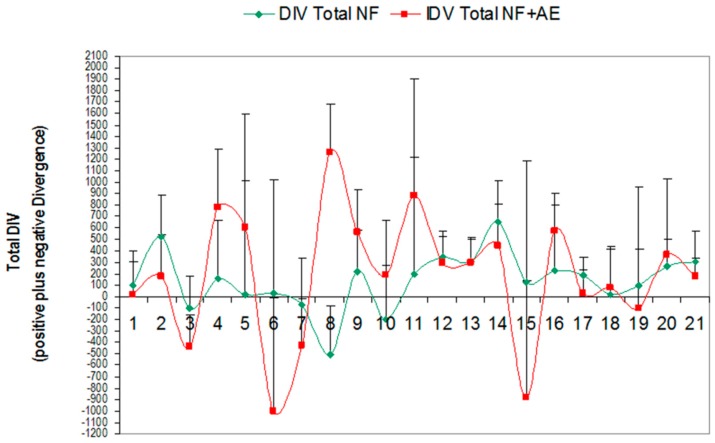
Total divergence (DIV) in trained bruxistic patients during 21 sessions with *Origanum majorana* essential oil (red: Total NF + AE) as compared levels in bruxistic patients who did not smell this fragrance during 21 NO training (green: Total NF). NF (ST) + AE: Divergences or total cerebral activity (DIV) in trained buxistic participants (with high “intrinsic stress”) during 21 NO sessions; they smelled *Origanum majorana* essential oil (*n* = 12 patients, 120 saliva samples, red color). (AE): patients exposed to *O. majorana* during 21 NO sessions. DIV Total (NF–ST): divergences or total cerebral activities (DIV) reached by training buxistic patients (with high “intrinsic stress”) in 21 NO sessions (without exposure to *O. majorana* essential oil; *n* = 12, 120 saliva samples, green). This figure shows the Divergence during 21 neurofeedback (NO) sessions (total: positive and negative) ± variance in bruxistic patients (with high “intrinsic stress”) that smelled *O. majorana* (red color) and participants who did not smell this fragrance (green color) in 21 NO sessions (21 × 12 = 252 values for pre-training plus 252 values for post-training sessions; total: 504 data).

**Figure 2:**
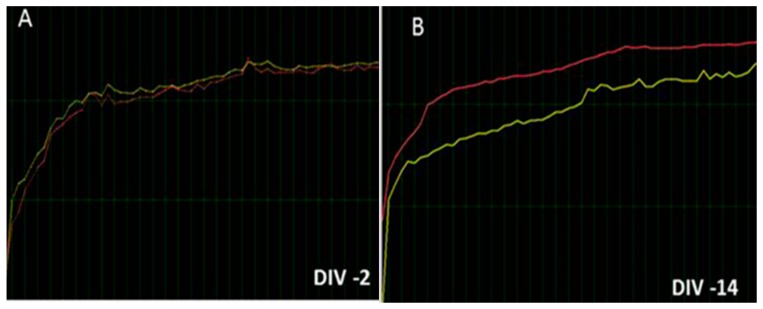

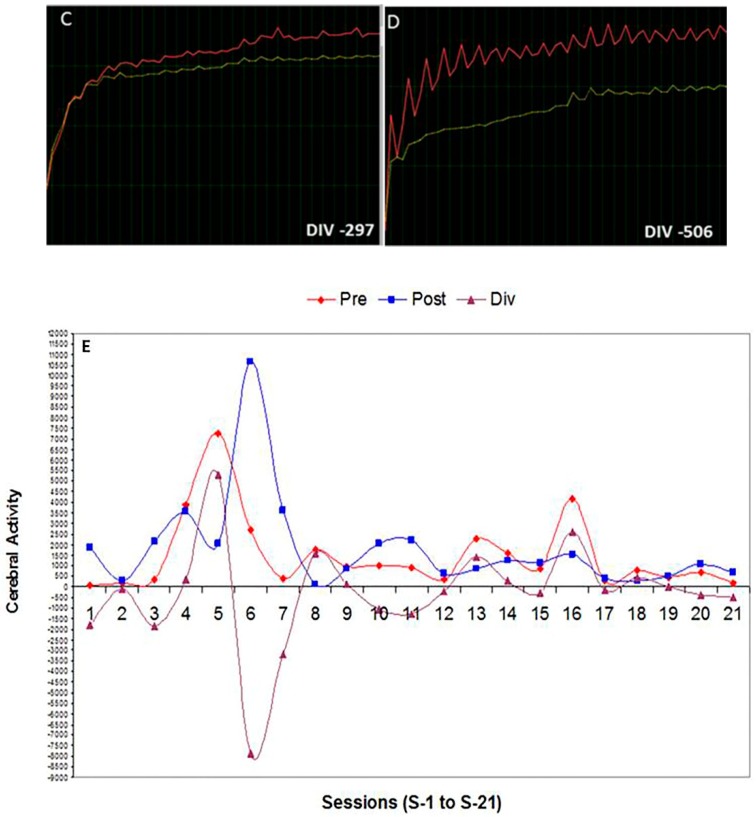
(**a**,**b**). Representative examples of cerebral activities (DIV) in bruxistic patients at pre-training (PRE: yellow) and post-training (POST: Red color) after 21 NO training sessions. (**a**,**c**) indicate representative cerebral activity levels (total divergences) in bruxistic patients with high “intrinsic stress”. The intrinsic stress is the experienced (perceived) stress by patients. Figure 2c shows DIV in these bruxistic patients after *Origanum majorana* exposure during 21 NO sessions (Figure 2c); note the lowest divergence values are seen in Figure 2 (**c**,**d**) (DIV: −297, −506); The Figure 2 (**a**,**b**) shows divergences close to zero (DIV: −2, −14 DIV), which means cerebral activity at pre-training stage (PRE: yellow) were close to post-training values (POST: red). These divergences (DIV) can be positive or negative depending on whether cerebral activity at pre-training was higher or lower than in the post-training level (POST, Figure 2e). DIV is the difference for cerebral activity in the PRE-training minus post-training and is considered an index of “brain efficacy” by NO training; thus, it reflected the efficacy of “brain regulation” after several NO training sessions. A negative DIV or one close to zero suggests a better cerebral state. Figure 2 (**e**) Divergences (DIV) fluctuate during 21 training sessions in bruxistic patient who have “high intrinsic stress” at post-training (POST: blue line), the pre-training values are shown in red (PRE: red line) and the difference (purple) represents divergence (DIV) parameter, which fluctuates reaching positive or negative values depending on whether cerebral activity found at pre-training was higher or not than the post-training values (POST).

**Figure 3:**
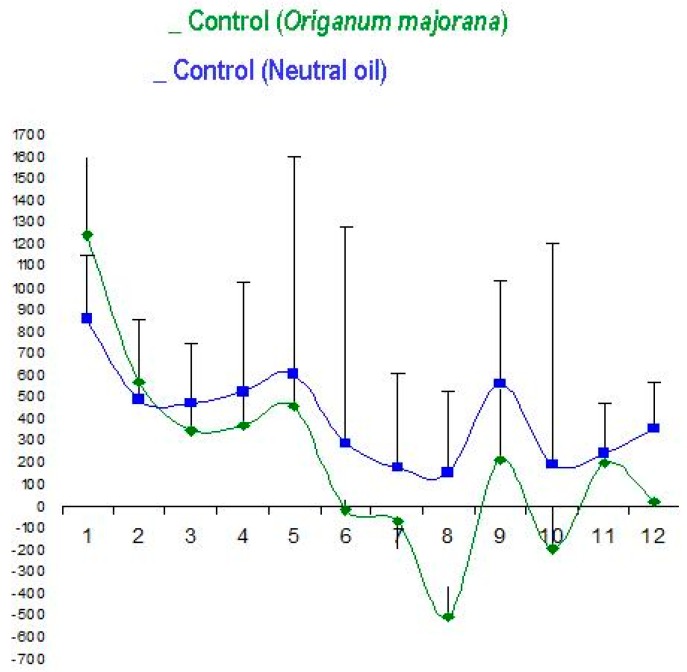
*Origanum majorana* essential oil exposure (green color, *n* = 5) did not affect total divergences (DIV) in control subjects exposed to *O. majorana* (12 NO sessions) as compared to placebo-treated patients (control subjects without stress); these placebo control subjects smell 1% neutral essential oil (blue colour, *n* = 5, *p* > 0.05, n.s) impregnated in nasal filters in 12 NO sessions.

Each NO training session takes 30 s at pre-training (PRE) and takes aproximately 34 min at post-training (POST) per patient. Cerebral activities were measured by the divergence (DIV) term. DIV is the difference between their cerebral activities at pre-training (PRE) minus the post-training level (POST). The total DIV was positive or negative depending on whether brain activity was higher or lower at pre-training than their respective post-training level in each training session (total: 21). MPO activity was measured in six NO sessions (S1, S6, S12, S18, S21) because NO changes are expected to occur within six NO training sessions according to Cabaña-Muñoz et al., 2016; this is the only paper that evaluates NO (neurofeedback neurotechnology) effects in patients [43]. All patients filled the Perceived Stress Scale questionnaire (PSS) to evaluate their stress level in the Cohen scale before/after 21 consecutive NO sessions (*n* = 24 participants) [32]. These PSS scores were compared between NO-trained bruxistic participants (with high “intrinsic stress”) without/with *Origanum majorana* essential oil exposure during 21 sessions. Their brain activities were compared with the initial NO session-1 (S-1). This means that 252 data of cerebral activity (12*21 sessions and 504 determinations were analyzed here, Figure 1, Figure 2 and Figure 3). Patients smelled 1% *Origanum majorana* essential oil impregnated into nasal filter during NO sessions. 

#### 2.3.2. Inclusion Criteria

The subjects completed a questionnaire on bruxism and underwent intraoral examination by an qualified dentist in order to detect possible eroding facets. They are 18–50 years old and 55% of the selected patients are women; they have a good general healthy state, including the mouths of bruxistic patients. All selected participants signed the written consent informed paper. Patients were included according their reached Q1–Q3 scores [44]. All the questions were evaluated using a 5-point rating scale: 0) never, 1) hardly ever, 2) occasionally, 3) fairly often, and 4) very often. The Nakayama questionnaire contains three questions (Q1, Q2, Q3) as follows:

Q1 In the last 3 months, has it been pointed out to you that you make a tooth grinding sound during sleep?
Q2 Do you experience orofacial jaw muscle fatigue or pain when you are awake?
Q3 When you are concentrating on something, or during work, do your upper and lower teeth

Nakayama et al., 2018 [44] have described these Q1–3 items; participants with a score ≤ 2 (never, hardly ever, and occasionally) were assigned a value of “0 (low frequency),” whereas those with a score ≥3 (fairly often and very often) were assigned a value of “1 (high frequency).” When either Q1 and Q2 scored 1, it indicated a strong possibility that the sleep-related signs (SBRS) were excessive. When Q3 was scored as 1, it indicated a strong possibility that the awake bruxism-related signs (ABRS) were excessive. The percentage of bruxistic patients were evaluated in the present study following these items [44].

Furthermore, during the intraoral examination, we selected bruxistic patients with zero score of Periodontal Index, which means absence of periodontal disease or inflammation in the present study (Periodontal Index of Community, WHO, 1997, Federation Dentaire Internationale). Thus, patients with values zero (absence of signs) were selected here.

In addition, we included bruxistic patients with high “intrinsic stress” when they reached values between 19–23 (moderate stress) for the Stress item (DASS-42 scale). This “intrinsic stress” is the current perceived stress experienced by patients. Scores for controls (without stress) are between 0–9 for the stress item (DASS-42) [45].

#### 2.3.3. Exclusion Criteria

We have excluded patients who have metabolic diseases (diabetes, metabolic syndrome, liver/kidney disease, systemic inflammation, lupus/autoimmune disease, thyroid disease, adrenal disease, Cushing syndrome, tumors, or neurological/psychiatric diseases (4th Edition, DSM IV). We did not enrolled patients who have brain disabilities or those suffering PSTD (post-traumatic stress disorder). In addition, participants who take regular medication (stimulants, anticonvulsants, antidepressant or psychiatric/bipolar drugs), chelators or antioxidant/anti-inflammatory supplements were not considered here. We excluded patients with periodontal diseases since periodontal disease is an infectious-inflammatory condition and we also excluded those with gingivitis or bacterial plaque [46]. We also excluded patients with orthodontic devices since increased MPO activity was detected in gingival crevicular fluid and whole saliva after fixed orthodontic appliance activation in patients [25]. The bruxism cannot cause periodontal disease *per se* in patients [47].

Finally, the influence of inflammation on periodontal disease could affect MPO activity, whereby patients with these characteristic were excluded in the present study [48]. These criteria are deep of sonage higher than 3 mm, loos of bone (radiography), possible bleebing and dental movility [48]. Periodontal explorations were done using a number 5 mouth mirror (Hu-Friedy^®^, Madrid, Spain) and OMS-sonde (PCP11 5 B, Hu-Friedy^®^, Madrid, Spain). The correct diagnostic of periodontal disease are based on several parameters such as visual exploration (palpation), presence of dental calculus, radiographic evaluation, dental movility, oclusal exploration (pathological eroding facets) [49,50,51,52].

Furthermore, during the intraoral examination, the Community Periodontal Index (CPI) was measured here (WHO, 1997), as a modification of the CPI of treatment needs (Federation Dentaire Internationale two-digit notation). The assessments covered the following six teeth or pairs of teeth (i) 16 and 17; (ii) 11; (iii) 26 and 27; (iv) 36 and 37; (v) 31 and (vi) 46 and 47. For each tooth, the depth of the gingival pocket was measured using a WHO-type probe, and the evaluation was then coded as follows: 0: no signs; 1: hemorrhage present; 2: dental calculus present; 3: gingival pocket 4-5 mm depth; and 4: gingival pocket of at least 6 mm depth. Thus, we selected patients with 0 scores, which means no signs. Thus, we excluded patients with values between 1–4, which are indicative of periodontal disease.

### 2.4. Depression, Anxiety and Stress Scale (DASS-42)

The DASS scale is a 42-item self-reported inventory that evaluates three factors: depression, anxiety, and stress by the Depression, Anxiety, and Stress Scale in patients (DASS-42) [45]. We used the stress item only (DASS-42 scale) in order to identify “intrinsic stress level” as moderate/high in patients. This inventory scale evaluates their physical anxiety (fear symptomatology) and mental stress (nervous tension and nervous energy), which represent two distinct domains. This screening and outcome measure also reflects the patient’s condition over the previous seven days. The normal ranges for the stress item are between 0–14 for controls (without stress) and bruxistic patients reached values between 19–25 here (*p* < 0.05 vs controls without stress). Unstressed controls have values between 0–14 for the Stress item (DASS-42 scale).

We selected bruxistic patients with “intrinsic stress” with scores between 19 and 25 for the Stress item (DASS-42 scale), which means they have moderate/high stress. The ranges for the Stress item are as follows (DASS-42 scale, Table 3):

### 2.5. The Perceived Stress Scale (PSS)

The PSS evaluates the stress degree in the recent month of life by testing 12 different items with five levels of intensity (Cohen scale); these Cohen scores are zero (never), 1 (almost never), 3 (sometimes), 4 (often) and 5 (very often). We compared PSS scores between NO trained-bruxistic participants with high “intrinsic stress” exposed (*n* = 17)/unexposed (*n* = 14) to *Origanum majorana* essential oil exposure during 21 NO training sessions (*n* = 17). In addition, divergences were compared between trained bruxistic patients with “high intrinsic stress” and untreaned bruxistic participants (with high “intrinsic stress”, *n* = 7). The total PSS score is the inversion of Cohen scores reached at 4, 5, 6, 7, 9, 10, 13 items as follows (0 = 4, 1 = 3, 2 = 2, 3 = 1, 4 = 0) and the summatory scores for all 14 different items. The higher scores predicts stress level according to the Cohen scale [32]. Results were expressed as percentage of untreaned (NO) bruxistic patients with high “intrinsic stress”.

### 2.6. NeurOptimal Technology: A Global Neurofeedback Technology that Measures Divergence (An Index of Cerebral Activity Capable of Predicting Brain Stability)

NeurOptimal^®^ (NO, a version of global Neurofeedback neurotechnology is a more advanced version of biofeedback and non-invasive technology (Zengar Institute, Toronto, ON, Canada). NO operates using electroencephalography (EEG) records to modulate brain activity during each training session from a global view point. The participant uses visual and auditory information presented by NO technology, which tries to “reorganize brain activity”. When the brain is performing fluidly, NO plays music but if the brain activity begins to become inconsistent or less smooth the music and image (fractals on the screen) are momentarily interrupted. The interruption gently cues the brain that it is not performing optimally. The software dynamically controls the patient feedback using non-linear statistics to calculate the precise timing that feedback is given at that exact moment. The feedback is given in the form of a pause in the music that is being listened to and a momentary hesitation of the fractal image appears on the screen of the computer. This means that brain disturbances are associated with music interruption during pre (PRE) and post-training sessions (POST). All of the learning happens outside of conscious awareness. As the brain begins to operate more efficiently, NO adjusts itself automatically, individualizing the training microsecond by microsecond to improve brain functioning. The participant must avoid moving large muscles or clenching their teeth. The primary feedback is auditory and the visual feedback is supportive during the NO training session.

NO client hookup consists of silver electrodes, applied to the subjects’ears and scalp, centered between the ear and the crown of the head on the bony ridge (Central points, cortex C3 and C4). The electrode sensors pick up the brains electrical signal and send it down a conductance wire to the Zengar Z-amp™ (Zengar Institute, Toronto, ON, Canada). This Z-amp™ cleans line noise and amplifies the brain wave signal. The left and right brain wave signals are separated by the computer software into their component frequencies and intensities. This continuous data set is analyzed using non-linear dynamical maths and statistics in order to determine when brain and nervous system enter into an area of “unstable” operation and feedback is given instantly within milliseconds. For more details on NO functioning, consult Cabaña- Muñoz et al., 2016 [43].

Neurofeedback (NO) training measures the total electrical activity for pre-/post-training sessions (wave cycles per second). Divergence (DIV) reflects the efficacy of training in terms of cerebral waves activities. DIV is the difference between brain activities detected at pre-training (PRE) minus the post-training (POST) level in each NO training session. DIV is positive or negative depending on whether cerebral activity at pre-training (PRE) is higher or lower than the respective post-training value (POST). The lower DIV, reflect the more stable the patient’s nervous system is at that precise training moment (Figure 1 and Figure 2). Thus, DIV can reflect “auto-plasticity” and cerebral activation after NO training in patients. Progress is not linear, meaning that the DIV numbers do not go down in a straight line in an orderly fashion. When DIV levels are higher, this means the information has not been integrated yet. Conversely, negative DIV suggests the information has been integrated at that particular training session. However, if cerebral activity at post-training (POST) are close to pre-training (PRE) value or higher, this means the information is being progressively integrated during the NO sessions until a final calm state is reached.

#### Neurofeedback (NO) Training Session

All brain activities were evaluated at pre-training (PRE) and post-training (POST) in 21 NO sessions. Each session takes 34 aproximately min for patient. Each NO session takes 30 s as pre-training (PRE, 15 s with open eyes and 15 s with closed eyes) plus another 33 min and 30 s in the post-training phase (POST). All participants begin the post-training phase immediately after concluding pre-training (PRE) without interruption. NO training was done twice a week (from 8:00 to 11:00 a.m.) and all bruxistic participants concluded their NO training within 120 days (2 sessions/week).

## 3. Results

The *Origanum majorana* essential oil exposure during NO training in healthy volunteers (non-bruxistic participants) induced a moderate elevation in brain activity in post-training sessions (POST) as compared to healthy volunteers (without smelling this odor) in a serie of 10 NO sessions. Repeated analysis of variance (ANOVA) reflect a lack of effect on total cerebral activity (DIV: positive and negative) according to Greenhouse–Geisser data [43].

### 3.1. Effect of Global Neurofeedback (NO) Overtraining during 21 Sessions on Total Divergence (Brain Activity) in Bruxistic Patients that Smell Origanum majorana Essential Oil

Kruskal–Wallis analysis reflected that 21 sessions of NO training did not significantly change subject’s total brain activity values (DIV: positive and negative) in bruxistic patients independently of *Origanum majorana* essential oil treatment during training (H (1, 20) = 1.12; *p* = 0.33; n.s). However, the levels of cerebral activity was progressively lower from initial training session until the last one in these bruxistic patients that smelled *Origanum majorana* essential oil in 21 sessions. In fact, after 21 NO consecutive post-training sessions (POST), these bruxistic patients that smelled this fragance (during training) progressively reduced their brain activity at certain sessions as compared to levels in their initial training session 1 (S-1, *p* < 0.05, data not shown).

### 3.2. Origanum majorana Essential Oil Has not Effect on NO Training Sessions in controls (without Stress) as compared to Placebo-Treated Patients with a Neutral Oil in Controls (without Stress)

The controls subjects (without stress) exposured to *Origanum majorana* essential oil during 12 NO sessions did not regulate cerebral activity (total DIV: positive and negative) as compared to placebo-treated controls (without stress). There are no difference on DIV between both controls in 12 NO sessions (*p* > 0.05 n.s, Figure 3).

The table indicates the percentage of patients who occasionally experienced bruxism-related signs (see Table 4). The subjects completed a questionnaire on bruxism and underwent intraoral examination by an expert; 62% of bruxistic positively answered to the present Q3 item following the Nakayama study [44]. As the frequency of bruxism is also judged by questionnaires, research based on objective evaluation methods, such as electromyograms, is necessary in future. All percentages are indicated in the Table 5.

### 3.3. Effect of Neurofeedback Training (NO) and/or Origanum Majorana Exposure during NO Sessions on Myeloperoxidase (MPO) Activity

**Figure 4:**
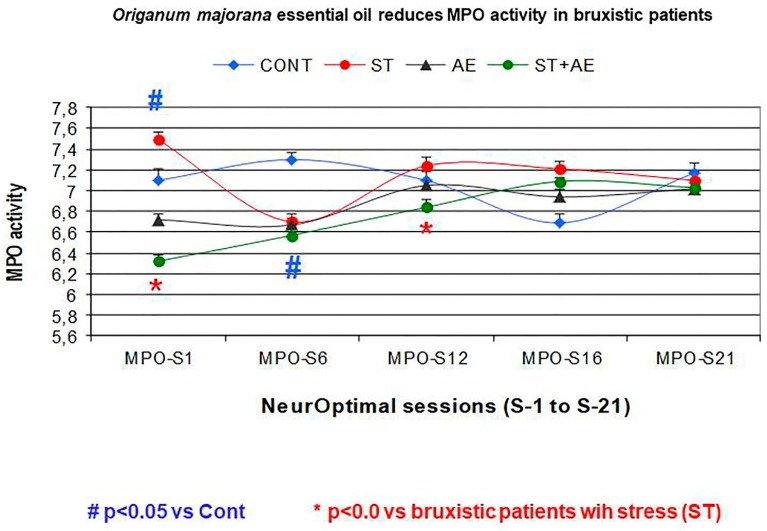
Effects of NO training and/or *Origanum majorana* essential oil exposure (AE) on saliva MPO activity in bruxistic patients at sessions 1, 6, 12, 16, 21. # *p* < 0.05 vs Cont. * *p* < 0.05 vs bruxistic patients with “intrinsic stress” ST. Control (blue): control subjects (without stress) who are not trained with NO. AE: non bruxistic participants (Controls) that smelled a 1% *Origanum majorana* essential oil impregnated into nasal filters. ST (Bruxistic): trained bruxistic patients with “high intrinsic stress” (ST) during 21 NO sessions but exposure to *Origanum majorana* essential oil. ST (Bruxistic)-AE: Bruxistic patients with “high intrinsic stress” who smelled 1% *Origanum majorana* essential oil impregnated in nasal filters during 21 sessions of NO training.

Enzymatic MPO activity was assayed in 500 saliva samples (*n* = 104 patients following the inclusion criteria in study groups). The repeated ANOVA revealed a significant effect on MPO activity in bruxistic patients with “high intrinsic stress” expose to *Origanum majorana* essential oil during NO sessions (S1, 6, 12, 18, 21) by reducing their MPO activity as a consequence of NO training (F (1,4) = 11.21, *p* = 0.037; power: 0.53, Figure 4).

Mann–Whitney post hoc tests revealed lower MPO activity by NO training in trained-bruxistic participants as compared to control subjects (*p* < 0.05). Moreover, saliva MPO activity progressively decreased after 21 NO sessions in bruxistic patients as compared to untrained bruxistic patients (*p* < 0.05). In addition, NO training at both sessions-1 and session 12 decreased MPO activities. However, NO overtraining lead to habituation on MPO activity (Figure 4). After the post-training phase (POST), the MPO activity decreased in bruxistic patients completing 21 sessions as compared their pre-training (PRE) data and controls also (untrained NO patients and unexposed to *O. majorana*, *p* = 0.1, n.s; data non shown).

The MPO activity did not differ between control subjects (without stress) expose to *Origanum majorana* essential oil and placebo-treated controls with a neutral oil (without stress) in 12 NO sessions (*p* > 0.05, S1, 6, 12; data non shown).

All participants smelled 1% *Origanum majorana* essential placed into nasal filters (InspiraHealth©) by diluting with a neutral essential oil. This essential oil was administered during each NO training session (21 sessions). These data are average MPO values ± S.E.M (standard error of the mean).

### 3.4. Effects of Neurofeedback Training (NO) and/or Origanum majorana Exposure on Stress Levels in the Stress Perceived Scale (PSS)

**Figure 5:**
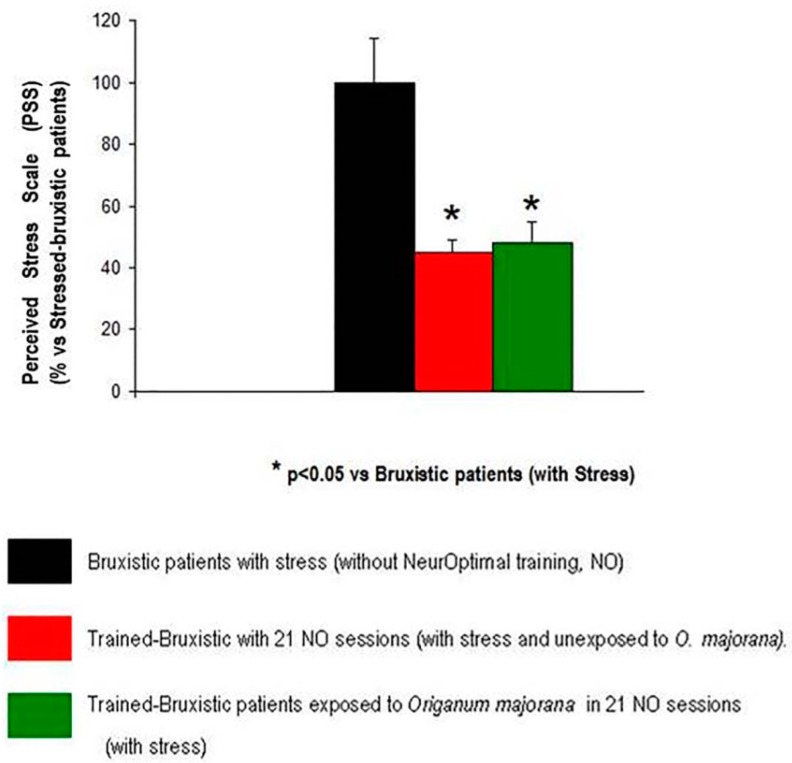
Bruxistic patients (with high “intrinsic stress”) unexposed/exposed to *Origanum majorana* during 21 NO sessions showed a decrease in their Stress Perceived Scale (PSS) percentage as compared to untreaned bruxistic patients.

The ANOVA have shown that 21 sessions of NO training affect Cohen PPS scores (F (2,36) = 6.14, *p* < 0.05) by reducing stress symptoms in bruxistic patients. The Bonferroni test reflected lower stress scores after 21 sessions of NO in bruxistic patients (with high “intrinsic stress”) that smell *O. majorana* essential oil than untrained bruxistic patients (with “high intrinsic stress”, black color). However, the PSS score did not differ between *Origanum majorana*-bruxistic trained patients (with high “intrinsic stress”) as compared to bruxistic patients unexposed to this fragance during 21 NO sessions (red color, *p* > 0.05, n.s). All patients answered the PSS scale after the NO training and results were expressed as a percentage of untreaned bruxistic patients (without NO training, black color; percentage of stress ± S.E.M: Figure 5).

## 4. Discussion

The pharmaceutical industry has investigated new antioxidants with a view to their application in dentistry [18,21,22,23]. The studies of Bracha and also Gungormus and Erciyas distinguish three emotional disorders, which are accompanied by occluso-muscle disorders, excessive experienced stress, depression, neurosis/phobias or anxiety in bruxistic patients [53,54].

There is no effective therapy against bruxism as yet except benzodiazepines treatment and relaxation techniques. It had been proved that compulsive, controlling, and aggressive persons are more vulnerable to develop bruxism [55]. Our findings suggest that repeated NO training during 21 consecutive sessions decreased stress symptoms in bruxistic patients. In addition, *Origanum majorana* essential oil inhalation during NO training seems to regulate their cerebral activities until they eventually reach a calm state. Thus, this essential oil seems to have an anxiolytic effect in bruxistic patients and could be a safe alternative for benzodiazepines treatment without dependence. *Origanum majorana* essential oil inhalation reduces saliva MPO activity after the first NO (S1) session, although NO overtraining leads to habituation according to MPO activity levels. Divergences (DIV: cerebral activities) fluctuate over the 21 NO sessions as a consequence of repeated training. In a dental clinic, the use of essential oils can reduce psychological stress and may prevent anxiety-related behaviors in the patients [16,56,57], including bruxistic patients. In fact, MPO activity decreased after NO training in the first session (S1), which could be attributable to the high terpenoid content (including tuyhanol) of *Origanum majorana* essential oil. Terpenoids can diffuse to the olfactory bulb and the limbic system, with beneficial effects in bruxistic participants with “intrinsic stress” who smell *Origanum majorana* fragrance during the 21 NO sessions. The lower MPO activity found at session 1 agrees with the decreased MPO activity reported in neutrophils from equine after *Sylibum marianum*-treatment in vitro [58]. However, there was a lack of effect on the global MPO activity after 21 training sessions in our patients, which may be explained by the habituation of MPO levels as a consequence of repeated training.

Neurofeedback training is effective as a measure of electrophysiological activity in cortical areas [35,36,37,38,39,40,41]. In fact, after 21 sessions NO training decreased stress levels in bruxistic patients who also smelled *Origanum majorana* essential oil during the sessions. However, patients’ cerebral activities fluctuated, enhancing desired electro-cortical activity and suppressing undesirable activity in the following NO sessions. These observations suggest that fluctuations in cerebral activity (DIV) are necessary before reaching a final “calm state” from repeated NO training. As the brain begins to operate more efficiently, NO adjusts itself automatically in response to the brain’s activity, individualizing the training microsecond by microsecond. The brain uses this information and reduces or increases its activity as a consequence of repeated training. The synergic NO training in bruxistic patients who smell *Origanum majorana* essential oil during training session may enhance their cognitive abilities and reduce stress level. Interestingly, *Melissa officinallis* has beneficial effects in bruxistic children without affecting their electromyographic recordings [19]. These findings in bruxistic children agree with the lack of global effect on divergence (brain activities) observed by us in the present study. The percentage of stress reduction we have observed in bruxistic patients in those that smell *Origanum majorana* fragance during NO training agrees with the relaxation state reported in patients who visited a dental clinic after *Lavander* or *Rosmarinus officinalis* essential oils were spreading in the waiting room [57,59,60,61,62,63,64].

So far, MPO can identify inflammatory responses in patients carrying orthodontic devices. The decrease in MPO activity observed in bruxistic patients after NO training at session-1 is not attributable to early stages of periodontal disease or gingivitis. In fact, these selected patients did not have signs of gingivitis or oral inflammation and were not carring brackets/orthodontic devices. We can not discount the possibility that bruxistic patients are more susceptible to stress by increasing MPO activity than bruxistic participants without stress. Increased MPO activity has been demonstrated in 20 patients who had orthodontic devices to treat different levels of dental crowding [25]. MPO activity could indirectly reflect stress and these bruxistic patients shows a rise in MPO activity at 2 h after crowding; however, their MPO activities reached levels were similar to controls at 7 and 14 days after activating orthodontic appliances [25,58]. As bruxistic patients did not carry orthodontic devices in the present study, their higher MPO activity could indirectly reflect stress. In addition, bruxism cannot cause periodontal damage per se in patients [47].

The Greenhouse–Geisser analysis for repeated ANOVA failed to shown a significant global effect on total cerebral activity (DIV) in bruxistic patients after 21 NO sessions. However, there was a significant effect on divergence (the average from session-1 to session-21) in bruxistic patients who were exposed to *Origanum majorana* after 21 NO training sessions (mean DIV: −580 ± 130) compared to those unexposed to this fragrance during the study (mean DIV: −130 ± 85, *p* < 0.05). Concurrent to NO training, the stress level decreased in these *Origanum majorana*-treated bruxistic participants after 21 NO sessions; this observation suggests that *O. majorana* essential oil enhances the anti-stress effect of NO training in bruxistic patients. Finally, bruxistic patients decreased their stress levels after repeated NO training more than untrained bruxistic participants as reflected by their respective Cohen stress scores (PSS test). However, *O. majorana* exposure during repeated NO sessions did not affect SPS stress scores as compared to levels in bruxistic patients unexposed to this essential oil during NO training.

Colectively, anxiolytic effects were demonstrated in bruxistic patients exposed to *Origanum majorana* essential oil during 21 NO training sessions. After NO training sessions, the bruxistic patients showed reduced stress and *Origanum majorana* exposure during NO training had regulated their cerebral activities in an appropriate way (divergence). The neurofeedback technology induces beneficial effects against depression (a very stressful condition), autism, attention deficit/hyperactivity disorder, Alzheimer disease, ischaemia and several brain disabilities [65,66,67,68,69,70,71]. The aromatherapy field supports the use of essential oils in these CNS pathologies [69]. Finally, NO technology measures global cerebral activity in the cortex by electrophysiological recording in patients. Currently, essential oils are spreaded in a waiting room (dental clinic) by diffusion given its beneficial effects in patients [60]. However, this feature could affect certain susceptible patients (i.e., (MCS): Multiple Chemical Sensitivity). The vehiculization of essential oils (*Origanum majorana*) impregnated into nasal filters do not harm these susceptible patients but aromatheraphy may provoke them some adverse effects if oils are spreaded in the waiting room.

## 5. Conclusions

NO training is an alternative neurotechnology capable of reducing stress in bruxistic patients and *Origanum majorana* inhalation (impregnated into nasal filters) during training sessions enhanced these anxiolytic effects in compliance with its antioxidant and anti-inflammatory properties. In fact, MPO activity significantly decreased at session-1 after NO training in bruxistic participants. This pilot study should be repeated with a larger sample size to confirm that MPO activity is a stress marker in bruxistic participants. As these trained bruxistic patients showed decreased scores in the stress perceived scale (PSS) after NO training than in trained bruxistic participants not exposed to the fragrance during NO training, we can assume *O. majorana* has anti-stress properties. However, the PSS stress scale did not differ between trained bruxistic patients after 21 NO sessions with/without *Origanum majorana* exposure. Colectively, the *Origanum majorana* essential oil enhanced the anti-stress effect of NO repeated training in bruxistic patients. Based on these observations, the use of NO technology could be extended to reduce stress in neuropsychiatric diseases or phobias (71).

## Figures and Tables

**Table 1:** Study groups.

Study Groups Total. 104 Participants and 500 Saliva Samples for Mieloperoxidase (MPO) Determination	*n*	Saliva Samples
(a) Trained Bruxistic participants (with high “intrinsic stress”) who smell *Origanum majorana* (AE) essential oil during 21 NO sessions.	12	120
(b) Trained Bruxistic participants (with high “intrinsic stress”) who do not smell *O. majorana* essential oil during 21 NO sessions.	12	120
(c) Bruxistic participants (with high “intrinsic stress”) without neurofeedback training	30	120
(d) Unstreased and not bruxistic patients (without NO training)	5	50
(e) Controls (unstressed and non bruxistic patients) without NO training.	30	60
(f) Controls (without stress) exposed to *O.majorana* during 12 NO sessions	5	15
(g) Control (placebo) patients who smell a neutral oil during 12 NO sessions	5	15

**Table 2:** Inclusion criteria (study groups).

NeurOptimal (Trained Bruxistic Patients)		NeurOptimal (Trained Controls)	
+ AE		+ AE	
**Inclusion Criteria**	**Inclusion Criteria**	**Inclusion Criteria**	**Inclusion Criteria**
Bruxistic patients (*n* = 12, 120 saliva)	Bruxistic patients (*n* = 12, 120 saliva)	Control Subjects (*n* = 5, 15 saliva)	Control Subjects (*n* = 5, 15 saliva)
With stress DASS-42 > 16–25 (Stress item)	With stress DASS-42 > 16–25 (Stress item)	Without stress DASS-42 (0–14) (for the Stress item)	Without stress DASS-42 (0–14) (for the Stress item)
Bruxism test and underwent oral examination (a good healthy state)	Bruxism test and underwent oral examination (a good healthy state)	Without Bruxism (oral examination and a good healthy state)	Without Bruxism
Trained in NeurOptimal (NO): 21 sessions	Trained in NO (21 sessions)	Trained in NO (12 sessions)	Trained in NO (12 sessions)
Exposed to *Origanum majorana* essential oil during 21 sessions	They underwent 21 NO sessions without *Origanum majorana* essential oil	Exposed to *Origanum majorana* essential oil during 12 sessions	Exposed to *placebo (neutral oil) in* 12 NO
**Inclusion Criteria**	**Inclusion Criteria**	**Exclusion Criteria** **(for all groups)**	**Inclusion Criteria**
Bruxistic patients (*n* = 30, 120 salive)	Control Subjects (*n* = 5, 50 saliva)	Periodontal disease or gingivitis	Control Subjects (*n* = 30, 60 saliva)
With stress: DASS-42 > 16–25 (Stress item)	Without stress DASS-42 (0–14) (for the Stress item)	presence of bacterial plaque	Without stress: DASS-42 (0–14) (for the Stress item)
Bruxism test and underwent oral examination (a good healthy state)	Without Bruxism (oral examination and a good healthy state).	Orthodontic devices	Without Bruxism
Without NeurOptimal (NO) training	Without *Origanum majorana* essential oil exposure	the use of removable partial denture	Without NO training
			Unexposed to *Origanum majorana*

+ AE: patients who smell *Origanum majorana* essential oil.

**Table 3:** References for stress item (Depression, Anxiety and Stress Scale, DASS-42).

	Normal	Mild	Moderate	Severe	Very Severe
Stress	0–14	15–18	19–25	26–33	>34

**Table 4:** Percentage of bruxistic patients.

Questionnaire (Bruxistic Patients)	0 (Never)	1 (Hardly Ever)	2 Occasionally	3 Fairly Often	4 Very Often
**Q1**	75%	20%	5%	0%	0%
**Q2**	65%	15%	20%	0%	0%
**Q3**	28%	12%	22%	28%	10%

**Table 5:** Number and percentage of bruxim-related signs after binarizing the results.

Item	Never Hardly Ever Occasionally (0 Score)	Fairly Often Very Often (1 Score)
**Q1**	100%	0%
**Q2**	100%	0%
**Q1 = 1 or Q2 = 1 (high-SBRS)**	0%	0%
**Q3 (high-ABRS)**	62%	38%

SBRS: sleep bruxism; ABRS: awake bruxism.
